# A Belgian Serosurveillance/Seroprevalence Study of Diphtheria, Tetanus and Pertussis Using a Luminex xMAP Technology-Based Pentaplex

**DOI:** 10.3390/vaccines4020016

**Published:** 2016-05-10

**Authors:** Raissa Nadège Caboré, Denis Piérard, Kris Huygen

**Affiliations:** 1 National Reference Centre *Bordetella* and *Toxigenic Corynebacteria*, Service Immunology, Scientific Institute of Public Health (WIV-ISP, Site Ukkel), 1180 Brussels, Belgium; RaissaNadege.CABORE@wiv-isp.be; 2 National Reference Centre *Bordetella* and *Toxigenic Corynebacteria*, University Hospital VUB, UZ Brussels, 1090 Jette, Belgium; Denis.Pierard@uzbrussel.be

**Keywords:** seroprevalence, serosurveillance, pertussis, diphtheria, tetanus, multiplex

## Abstract

Serosurveillance and seroprevalence studies are an essential tool to monitor vaccine-preventable diseases. We have developed a magnetic bead-based pentaplex immunoassay (MIA) for the simultaneous detection of IgG antibodies against diphtheria toxin (DT), tetanus toxin (TT), pertussis toxin (PT), filamentous hemagglutinin (FHA) and pertactin (Prn). The in-house pentaplex MIA showed a good correlation with commercial ELISAs with correlation coefficients between 0.89 for PT and 0.98 for TT. Intra- and inter-assay variability was <10%. A total of 670 anonymized serum samples collected in 2012 in Belgian adults (ages 20–29.9 years) were analyzed. Geometric mean concentrations (GMC) were 0.2 (0.13–0.29) IU/mL for DT, 0.63 (0.45–0.82) IU/mL for TT, 3.9 (2.6–5.8) IU/mL for PT, 16.3 (11.7–22.7) IU/mL for FHA and 15.4 (10.1–23.6) IU/mL for Prn. Antibody concentrations were below the protective level of 0.1 IU/mL in 26.4% of the sera for DT and in 8.6% of the sera for TT. Anti-PT IgG concentrations indicative of recent pertussis infection (>125 IU/mL) were detected in 1.2% of the subjects. High anti-PT antibodies were not correlated with high antibodies against any of the four other vaccine antigens. This pentaplex MIA will be used for a new large-scale Belgian serosurveillance/seroprevalence study of diphtheria, tetanus and pertussis.

## 1. Introduction

Serosurveillance and seroprevalence studies are an essential tool to monitor vaccine-preventable diseases, as they can detect subgroups at risk and therefore deliver information that is often lacking in routine surveillance and vaccination coverage studies. The last age-specific serosurveillance data for measles, mumps, rubella, diphtheria and tetanus for Belgium, reported by Theeten *et al.* in 2011*,* concerned samples collected in 2006 [1]. In that study, protective antibody levels of >0.1 IU/mL for diphtheria were detected in >70% in all age groups <30 years. However, the seroprotection rate decreased steeply with age in subjects aged >30 years, to a minimum of 20% in those 55–59 years. Overall, the diphtheria seroprotection rate in the general population was suboptimal at 55.2%. For tetanus, only subjects aged >40 years were tested, and 90.7% were found to have a protective concentration level of >0.1 IU/mL [1]. Pertussis was not included in that study, and the last official report on whooping cough seroprevalence, published in 2003, analyzed samples collected in Flanders between April 1993 and February 1994 [2]. We have previously reported on anti-pertussis toxin pertussis toxin (PT) antibodies in 1500 anonymized leftover diagnostic samples, collected randomly during the second semester of 2012, by the laboratories of clinical biology of six participating Belgian centers, among ”healthy” asymptomatic adults aged between 20 and 39 years old [3]. Sixty-one (4%) of the sera had anti-PT levels indicative of an infection in the last few years, and another 61 (4%) had anti-PT IgG antibodies reflecting a recent pertussis infection. These results showed that also in Belgium, as in many other industrialized countries, a *Bordetella pertussis* reservoir is present in the adult ‘healthy’ population, which now represents the main source of infection for young infants. As serum samples were anonymized, it was not possible to exclude that the high anti-PT concentrations observed were the result of recent booster vaccinations administered in the context of a cocoon or pregnancy vaccination, although the latter strategy was not yet advocated in 2012 and the former applied only with little success. Furthermore, for the 2012 study, we only reported on antibodies against PT, but not against the other four antigens that are present in the booster vaccine (diphtheria toxin (DT), tetanus toxin (TT), filamentous hemagglutinin (FHA) and pertactin (Prn)). 

A number of studies have previously reported on the development of multiplex assays for the screening of antibodies against these five vaccine antigens [4,5,6,7,8], but commercial multiplex kits are not available in Belgium for this type of analysis. We therefore developed and validated an in-house magnetic bead-based pentaplex immunoassay (MIA) for the combined quantification of IgG antibodies against diphtheria, tetanus and pertussis. In order to secure antigen availability and future standardization, commercially available, highly-purified antigens were used. Because ELISA kits recommend a dilution of 1:100, we used this same dilution in the pentaplex MIA. The multiplexing capacity of the Luminex platform allows for a single small sample to be simultaneously tested against multiple antigens, which is advantageous over methods, such as ELISA, because the results are generated in one run, and rather than performing separate individual assays for each diphtheria-tetanus-pertussis (DTP) vaccine antigen, antibody concentrations for all antigens can be determined simultaneously with a single serum dilution. This not only greatly reduces time and labor, but also conserves sample volumes, which, in the case of pediatric samples, might be in limited supply; and it avoids the multiple freeze/thaw cycles that can alter antibodies. For example, with Luminex, a single dilution (1:100) of 10 μL or less of serum is sufficient to perform all five assays. In contrast, more than 50 μL of serum sample would be required to run five classical ELISAs. 

Here, we report on the development of the in-house pentaplex MIA and its use for the analysis of 670 serum samples (in the 20–29.9 years age category) of our 2012 study [3] for the presence of antibodies against the five vaccine antigens.

## 2. Materials and Methods

### 2.1. Antigens and Reagents

Purified toxoid from Corynebacterium diphtheriae DTxd (NIBSC 02/176) was purchased from the National Institute of Biological Standards and Control (NIBSC) (Hertfordshire, U.K.), toxoid from Clostridium tetani TTxd (T3194) from Sigma-Aldrich (Saint Louis, MO, USA), pertussis toxin (PT) from Statens Serum Institute (Copenhagen, Denmark) (Batch SSI 83151), filamentous hemagglutinin (FHA) from Kaketsuken (Kumamoto, Japan) and pertactin (Prn) from NIBSC (NIBSC90/654). Magnetic carboxylated microspheres (MagPlex^TM^-C) were purchased from Luminex Corporation (Austin, TX, USA). N-hydroxysulfosuccinimide (sulfo-NHS) and 1-ethyl-3-(3-dimethylaminopropyl)carbodiimide hydrochloride (EDC) were purchased from Pierce (Rockford, IL, USA). R-phycoerythrin (RPE)-conjugated goat anti-human IgG antibodies were obtained from Jackson ImmunoResearch Westgrove, PA, U.K.) (Jackson IR 109-116-098). Phosphate-buffered saline (PBS), pH 7.2, was obtained from Life Technologies (Gibco^®^, Grand Island, NY, USA). Bovine serum albumin (BSA), Tween 20, monobasic sodium phosphate (NaH_2_PO_4_), sodium azide (NaN_3_) and 2-(N-morpholino)ethane-sulfonic acid (MES) were purchased from Sigma Aldrich (Saint Louis, MO, USA).

### 2.2. Reference, Control and Validation Sera 

National Institute of Biological Standards and Control (NIBSC) standards for diphtheria antiserum (NIBSC Code 10-262, diphtheria antitoxin 2 IU/mL), for tetanus antiserum (NIBSC Code TE-3, 10 IU/mL) and for pertussis antiserum (NIBSC Code 06/140: anti-PT 335 IU/mL, anti-FHA 130 IU/mL and anti-Prn 65 IU/mL) were used to determine the IgG concentrations in IU/mL of an in-house reference standard consisting of a pool of sera, generated in the context of a study on maternal pertussis vaccination, *i.e.*, in women who received a tetanus-diphtheria-pertussis (Tdap) booster vaccination and showed high antibody concentrations to the five antigens [9,10]. As this pentaplex was also developed for a serosurveillance study of diphtheria and tetanus, we deliberately chose not to use sera from patients with acute pertussis infection, because the levels of DT and TT antibodies in such sera would not have been suitable for making a wide-range standard curve. A panel of 37 serum samples, tested in a DTP antibody screening study by classical ELISA, of vaccinated children with recurrent and/or invasive pyogenic infections (provided by Prof. Xavier Bossuyt, Laboratory Medicine, Immunology, University Hospitals, KU Leuven, Leuven, Belgium), was used for the validation of the MIA. 

### 2.3. ELISA 

The panel of 37 serum samples was first tested by ELISA. IgG antibodies against individual DTP vaccine antigens were determined using commercially available ELISA kits: Virotech/Sekisui^®^ ELISA kit (Rüsselsheim, Germany) for anti-DT and anti-TT antibodies, EuroImmune^®^ ELISA (Lübeck, Germany) for anti-Prn and anti-FHA antibodies and Virion/Serion^®^ kit (Würzburg, Germany) for anti-PT antibodies. The assays were performed according to the manufacturer’s instructions. Briefly, serum samples were diluted 1:100 and tested in duplicate. Optical density (OD) was read using the Bio-Rad ELISA reader. OD values within the linear part of the curve were converted to IU/mL by interpolation from a 4-parameter logistic (4-PL) standard curve of the reference serum and averaged. 

### 2.4. Conjugation of Capture Antigens to Microspheres

The coupling was performed at room temperature according to the two-step carbodiimide reaction protocol according to Luminex cookbook 2nd edition [11]. All washing steps were performed using a magnetic separator. The microspheres were vortexed vigorously for 20 s and then suspended in a sonicating water bath (Bransonic^®^, Branson Ultrasonic Corporation, Danbury, CT, USA) for 20 s in order to disperse bead aggregates. For each selected target, the optimal antigen concentrations was chosen according to Van Gageldonk *et al.* with minor modifications, to provide the highest signal-to-noise ratio and the most consistent and reproducible results [6]. Each antigen was covalently linked to a specific bead set: Prn (#12), FHA (#33), TT (#38), DT (#36) and PT (#72). Briefly, 12.5 × 10^6^ carboxylated beads were activated by incubation with 500 µL of activator buffer (0.1 M NaH_2_PO_4_, pH 6.2) containing 2.5 mg/mL EDC and 2.5 mg/mL sulfo-NHS for 20 min at RT in the dark under constant rotation. Then, the activated beads were washed three times and re-suspended in coupling buffer (50 mM MES, pH 5.0). Coupling was performed by adding 500 µL (antigen-to-bead ratios of 10 μg/12.5 × 10^6^ for all antigens) of the antigen solution to the pellet of activated microspheres and allowed to incubate for 2 h at RT in the dark and under constant rotation. After the coupling process, the beads were washed three times, re-suspended in blocking/storage buffer (PBS, 1% BSA 0.05% NaN_3_), counted and stored in the dark at 2–8 °C until use. 

### 2.5. Pentaplex MIA 

MIA was used to analyze a total of 670 anonymized left-over serum samples, collected in 2012 by six clinical chemistry laboratories in Belgium among healthy adults 20–29.9 years old [3]. The samples had been stored at −20 °C, were thawed once for MIA and frozen again the same day. All incubations were done at room temperature (20–25 °C) in the dark on a rotating shaker (set at 500–600 rpm) using black and flat-bottomed 96-well microplates (Greiner 655096). The serum samples were diluted 1:100 in assay buffer (PBS-3%BSA-0.1%Tween 20 pH 7.2). All washing steps were performed using a handheld magnetic plate washer (Millipore, Missouri, USA). Bead sets carrying different protein antigens were mixed by vortexing, sonicated and diluted in assay buffer (to a final concentration of 1.25 × 10^4^ beads per well (2500 beads/well for each type). A volume of 50 µL of serum sample, diluted in assay buffer (PBS-1%BSA-3%Tween 20 ), was mixed 1:1 with the beads and incubated for 1h. All samples were run in duplicate. The beads were washed three times with wash buffer (PBS-1%BSA). Next, 100 μL of a 1:200 dilution of RPE-conjugated goat anti-human IgG in assay buffer was added to each well, and the plate was incubated for 30 min under continuous shaking at room temperature. After a final washing step, beads were re-suspended in 100 μL of wash buffer and analyzed on a MAGPIX in combination with xPONENT Manager software (Luminex Corp., Austin, TX, USA). Each bead is identified by its signature fluorescent pattern and then analyzed for the mean fluorescence intensity (MFI) of the signal of the reporter antibody on ≥50 microspheres per set per well. The MFI is directly proportional to the amount of analyte (*i.e.*, antigen-specific antibody) bound to a given bead set. For each analyte, MFI was converted to IU/mL by interpolation from a 5-PL in-house standard curve of (log-log) transformed data for every bead region/standard. Using the default settings in this study, the xPONENT software gates all results to recognize double beads or aggregates of beads, so they can be excluded or retested as applicable. Dilutions of international reference standards and blanks (background) were included in each run to monitor assay reproducibility and precision. The MFI data were corrected for background (Bkg) levels by subtracting the background MFI signal from the sample MFI signal (MFI-Bkg). 

## 3. Results

### 3.1. Validation of the Pentaplex Immunoassay

**Figure 1:**
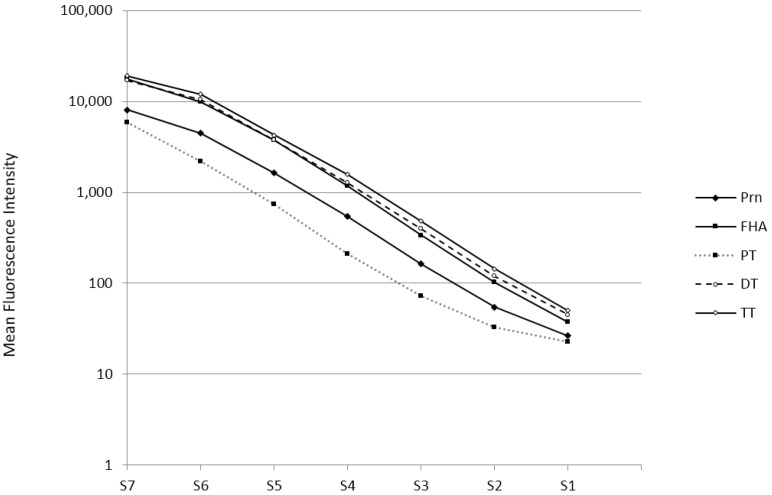
In-house standard curves for diphtheria toxin (DT), tetanus toxin (TT), pertussis toxin (PT), filamentous hemagglutinin (FHA) and pertactin (Prn) of seven four-fold serum dilutions, with serum (S)7 being the initial dilution of 1:100 and S1 being the last dilution of 1:409,600. The curves represent the mean fluorescence intensity of each serum dilution for each analyte on a log scale.

Bead sets were first evaluated separately in a monoplex using the NIBSC reference standards for diphtheria, tetanus and pertussis (Codes 10.262, TE-3 and 06/140, respectively) for calibration. Next, bead sets were mixed, and seven serial four-fold dilutions (from 1:100–1:409,600) of the in-house reference standard were tested in the pentaplex MIA. Standard curves for the five analytes from a representative assay run are shown in Figure 1. All curves showed similar parallel slopes and were linear over approximately seven four-fold dilutions. This range of the in-house standard is higher than for standards in commercial ELISA, hence avoiding the need for multiple sample dilutions. Using dilution curves of the international standard preparations, values of diphtheria-, tetanus- and pertussis-specific antibody concentrations of the in-house reference serum were determined: DT: 3.0 IU/mL; TT: 3.5 IU/mL; PT: 97 IU/mL; FHA: 292 IU/mL; and Prn: 1333 IU/mL. 

To assess the reproducibility of the pentaplex, intra-assay (samples tested three times within a run) and inter-assay variation (sample tested in three runs on three different days) was evaluated, and coefficients of variation (CV) were calculated for each measurement (Table 1). The lower limit of detection (LLOD) of each analyte was determined by interpolation of the 15 blank mean MFI value + 2 standard deviations (SD) in the pentaplex from the in-house reference curves and represented as the concentration (IU/mL) (Table 1). 

An intrinsic problem of the Luminex technology for serological assays is the finding that human sera may contain heterophilic antibodies that directly bind to the Luminex beads, resulting in a nonspecific background [12,13]. In our test, which uses magnetic beads instead of carboxylated microspheres, we observed only very low background values between 15 and 35 MFI, indicating that heterophilic antibodies did not influence the assay. Specificity was not tested separately, as we used highly purified antigens. In contrast, during the coupling procedure, we observed a loss of signal with high analyte exposure, probably due to the long-established hook effect as previously described by Amarasiri, *et al.* [14]. 

### 3.2. Validation of the Pentaplex on a Small Serum Panel

**Figure A1:**
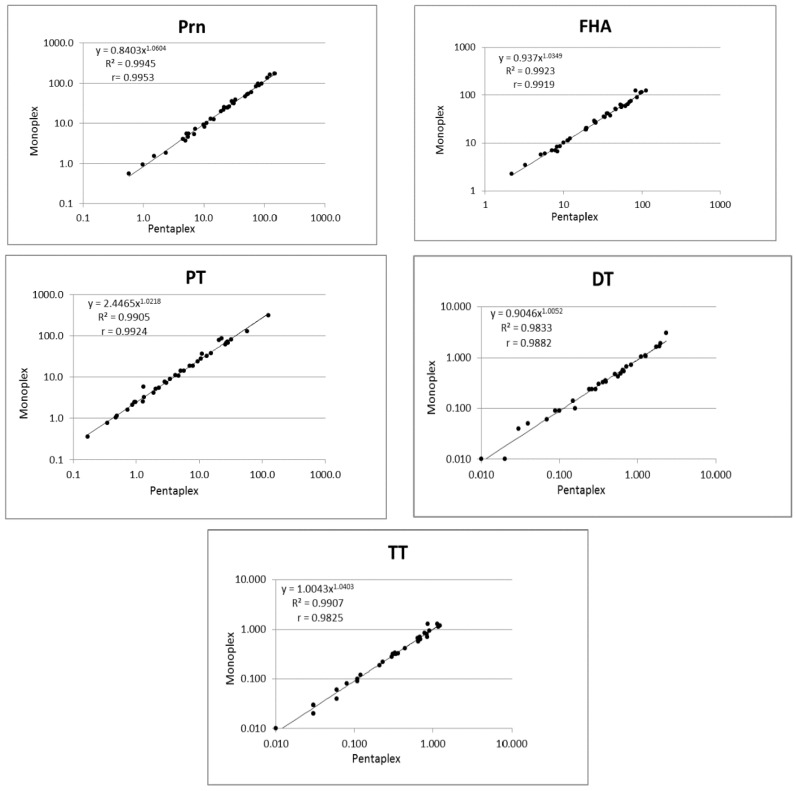
Correlation of anti-Prn, anti-FHA, anti-PT, anti-DT and anti-TT IgG concentrations (IU/mL) of a small serum panel (*n* = 37) as detected by monoplex and pentaplex assay. *R*^2^ represents the linear regression coefficient obtained using Microsoft Excel, and *r* represents the non-parametric Spearman correlation coefficient as calculated with GraphPad Prism Version 6 (*p* < 0.0001 for all five analytes).

Possible interference between the different bead sets was checked by comparing the MFIs detected by the monoplex MIA with those detected by the pentaplex MIA using the panel of thirty-seven serum samples, from a DTP antibody screening study. The results were analyzed by linear regression, and the Spearman correlation coefficients (*r* values) were determined. An excellent correlation ranging from 0.98 for TT to 0.99 for Prn was found between the monoplex and pentaplex MIA, and no evidence of bead interference between the different bead sets was found (Appendix Figure A1). In addition, the MFIs generated by the pentaplex MIA were similar to those generated by the monoplex assay. 

**Figure 2:**
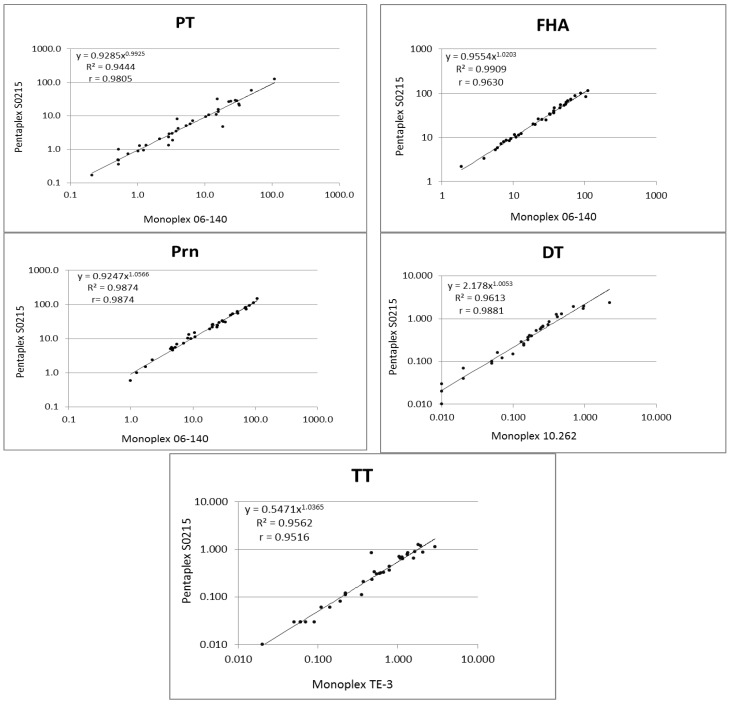
Correlation of anti-DT, anti-TT, anti-PT, anti-FHA and anti-Prn IgG antibodies detected in 37 serum samples using the pentaplex immunoassay (MIA) with the in-house standard and monoplex MIA using the international reference standards. Dots represent individual log-transformed antibody concentrations expressed in International Units per milliliter (IU/mL). *R*^2^ represents the linear regression coefficient obtained using Microsoft Excel, and *r* represents the non-parametric Spearman correlation coefficient as calculated with GraphPad Prism Version 6 (*p* < 0.0001 for all five analytes).

As shown in Figure 2, antibody concentrations of the 37 samples determined by the pentaplex/in-house reference standard correlated very well with concentrations determined by the monoplex/international reference standard.

**Figure 3:**
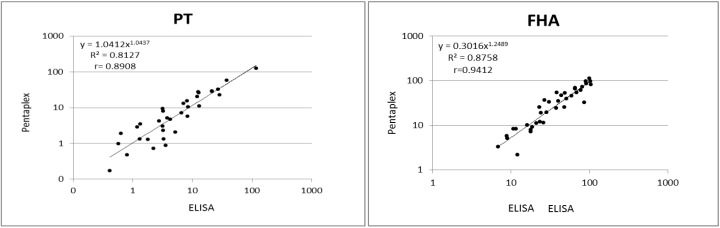

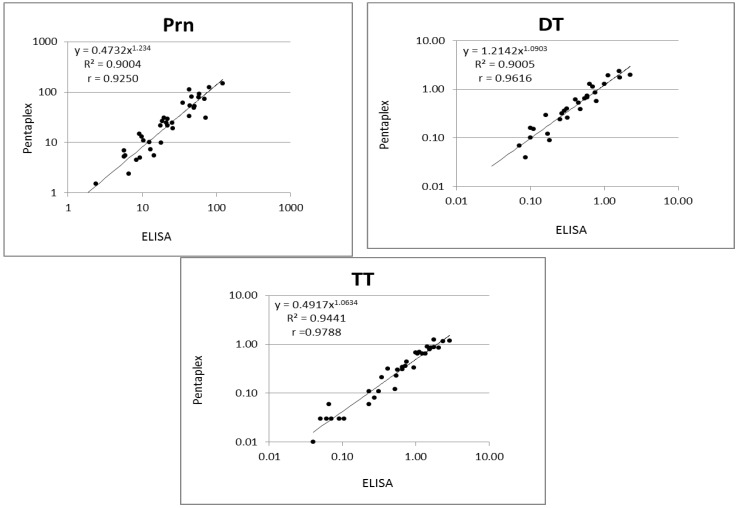
Correlation of IgG concentrations to DTP antigens obtained with pentaplex immunoassay (MIA) and commercial ELISAs. Serum samples (*n* = 37) were measured by both methods as described in the Materials and Methods section. Antibody concentrations in International Units per milliliter (IU/mL) shown after log transformation are shown for DTP antigens. *R*^2^ represents the linear regression coefficient obtained using Microsoft Excel, and *r* represents the non-parametric Spearman correlation coefficient as calculated with GraphPad Prism Version 6 (*p* < 0.0001 for all five analytes).

Finally, results obtained in the pentaplex using the in-house standard were compared to results previously obtained with commercially available ELISAs for all five antigens. Antibody concentrations determined by both methods were analyzed using linear regression analysis. The graphic display of the linear regression analysis *R*^2^ and Spearman correlation coefficient *r* is shown in Figure 3. The MIA results correlated very well with ELISA for all DTP antigens tested, with Spearman correlation coefficients between 0.89 for PT and 0.98 for TT. Van Gageldonk *et al.* [6] used in a previous study the same capture antigens in ELISA and in MIA and obtained a correlation between both tests of 0.98 for pertussis antigens and tetanus toxin and 0.95 for diphtheria toxin. 

Using Bland–Altman correlation analysis, ELISA-MIA ratios were 0.43 (SD 0.35) for PT, 1.52 (SD 0.85) for FHA, 1.21 (SD 0.66) for Prn, 0.96 (SD 0.31) for DT and 2.3 (SD 0.83) for TT.

### 3.3. Results of the Serosurveillance/Seroprevalence Study

We next proceeded to analyze by MIA a total of 670 anonymized left-over serum samples, collected in 2012 by six clinical chemistry laboratories in Belgium among healthy adults 20–29.9 years old. As a quality control, the international standard preparations were analyzed in parallel in the sixteen runs needed to perform the global analysis. The following mean values were determined: anti-DT (2 IU/mL): 2.97 IU/mL (6% CV); anti-TT (10 IU/mL): 7.01 IU/mL (18% CV); anti-PT (335 IU/mL): 315 IU/mL (6% CV); anti-FHA (130 IU/mL): 118 IU/mL (15% CV); and anti-Prn (65 IU/mL): 63 IU/mL (10% CV). Overall, the observed values were very close to the defined concentrations for the three pertussis antigens, but higher for DT and lower for TT. 

Pentaplex results for the five vaccine antigens, *i.e.*, DT, TT, PT, FHA and Prn, measured in each age group are shown in Table 2. GMC were 0.2 (0.13–0.29) IU/mL for DT, 0.63 (0.45–0.82) IU/mL for TT, 3.9 (2.6–5.8) IU/mL for PT, 16.3 (11.7–22.7) IU/mL for FHA and 15.4 (10.1–23.6) IU/mL for Prn. 

Based on the population distribution of Belgium in 2012 for this age category (Flanders 55.1%, Wallonia 32.3% and Brussels Capital region 12.6%), antibody concentrations were estimated to be below the protective level of 0.1 IU/mL in 26.4% of the sera for DT and in 8.6% of the sera for TT (Table 3). A total of 46 (6.8%) of the sera had anti-PT concentrations above 50 IU/mL, indicative of a pertussis infection during the last few years (Table 3). 

As PT-specific antibodies can also be induced by vaccination (albeit with a rapid decline), we analyzed the possible correlation of high PT antibody levels with antibody levels against the other four antigens. GMC and 95% upper and lower CI of anti-Prn, anti-FHA, anti-TT and anti-DT were determined for the 38 samples with anti-PT concentrations between 50 and 125 IU/mL and for the eight samples with anti-PT concentrations above 125 IU/mL (Table 4). Using Spearman non-parametric two-tailed correlation analysis of paired samples, no statistical correlation was found between anti-PT and either of the four other antibodies, indicating that the high anti-PT levels were almost certainly the result of the indication of a recent *Bordetella* infection, rather than of a recent vaccination. However, a statistically-significant correlation was found between anti-Prn and anti-FHA levels in the group with anti-PT levels between 50 and 125 IU/mL (*p* = 0.001). For the group with anti-PT levels >125 IU/mL, no statistical correlation was found between anti-PT and either of the four other antibodies either.

## 4. Discussions and Conclusions

The aim of this serological survey was to evaluate the seropositivity in 2012 for diphtheria and tetanus and the seroprevalence for pertussis of “healthy” Belgian adults aged 20–29.9 years old (born between 1983 and 1992), three infectious diseases against which vaccination has been widely implemented since the end of the 1950s. 

Based on the MIA results for anti-diphtheria antibodies in the age group 20–29.9 years, we estimated that only 73.6% of the subjects (adjusted according to the distribution of the Belgian population) showed sufficient antibodies to be protected. Confirming the Theeten study (at least for the male population), more diphtheria seronegative sera were detected in samples collected at the two centers from Brussels than from Flanders or Wallonia [1]. Demographic differences may lie at the basis of this difference, as Brussels Capital Region has a more cosmopolitan population (with many citizens of non-Belgian origin). Thanks to vaccination, diphtheria has almost disappeared in Western Europe; however, cases of *C. diphtheriae* related to exposure in endemic countries and of *C. ulcerans* of zoonotic origin are sporadically reported each year, and immunization against diphtheria remains important [15]. In 2012, one case of toxigenic *C. ulcerans* was reported in Belgium in a 72-year-old woman, who presented with a chronic leg ulceration. She was probably protected by her recent dTap booster vaccination, as she did not present systemic symptoms. Although zoonotic transmission could not be proven, this remained the most probable source of infection [16]. In March 2016, a fatal case of diphtheria occurred in Belgium, in a three-year-old girl, whose absent anti-DT antibodies confirmed the child had not been vaccinated [17].

For tetanus, the Theeten study evaluated only subjects aged >40 years, and found 90.7% to have protective antibody concentrations of >0.1 IU/mL [1]. In our present MIA study, we could calculate a very similar seroprotection level of 91.4% in subjects 20–29.9 years old. 

Global pertussis vaccination programs have been introduced with success, and approximately 84% of infants worldwide have received three doses of the diphtheria-tetanus-pertussis (DTP3) vaccine [18]. However, a decade after the switch from the whole-cell (wP) vaccine to the acellular pertussis (aP) vaccine, a resurgence has been reported in several industrialized countries, in all age categories. Whereas morbidity and mortality occur primarily in young infants who are not fully vaccinated [19], the majority of cases are found in adolescents and adults, in whom pertussis can be even more or less asymptomatic [20]. The present study enabled us to compare our MIA pertussis seroprevalence data with results previously obtained using a commercial anti-PT ELISA [3]. Pentaplex analysis showed that 6.8% of the sera had anti-PT concentrations higher than 50 IU/mL, indicative of a pertussis infection during the last few years. This percentage is close to the 8% we reported in the larger study on 1500 subjects 20–39 years old. 

The laboratory diagnosis of pertussis is based on the direct detection of the bacteria or their DNA by culture or PCR and by indirect anti-PT IgG serology [21]. Whereas PCR is most sensitive at the onset of infection, serology is particularly indicated for the diagnosis of pertussis in adults, who often present for medical care at a later stage. There is some variation in the threshold anti-PT values used in different studies for defining an acute pertussis infection. In 2004, Baughman, *et al.* proposed an anti-PT IgG level of 94 ELISA units/mL as the diagnostic cut-off point for recent *B. pertussis* infection in adolescents and adults with cough illness in the United States [22]. A recommendation from the EU Pertstrain group of 2011 indicated that for single-sample serology, IgG anti-PT antibodies below 40 IU/mL can be considered negative (not indicative of contact with *Bordetella* during the last few years), whereas levels above 100 IU/mL can be used as an indicator of recent contact with the bacteria [23,24]. We have used the value of 125 IU/mL, which was originally defined by De Greef *et al.* [25] and which we have also used as threshold in a study on prevalence, diagnosis and disease course of pertussis in adults with acute cough in primary care [26]. It must be mentioned that for clinical diagnosis, this cut-off value may even be on the low side. As an example, of the 325 serum samples that were tested in November 2015 at the National Reference Centre for *Bordetella* in Belgium from patients with cough symptoms and no history of recent vaccination, 40 (12.3%) had anti-PT IgG titers above 125 IU/mL, and the GMC of this group was 266 IU/mL. 

On the basis of our MIA data, an estimated pertussis incidence of 1200/100,000 in the age group 20–29.9 years could be calculated, which is much higher than the 2012 incidence of 7.6–10.8/100,000 of acute pertussis officially reported jointly by the Belgian National Reference Centre for *Bordetella* and the Sentinel Laboratories [27]. In a recent mini-review on 44 seroprevalence studies of pertussis performed in 23 countries, Barkoff *et al.* have shown that incidence rates obtained from seroprevalence studies indeed differ significantly from incidence rates in the notification reports and that in general, there was a significant underestimation of pertussis cases among whole populations [28]. Using the same Luminex xMAP technology for the detection of anti-PT IgG, a recent seroprevalence study of pertussis in 1067 individuals aged between two and 90 years old in rural Gambia found that 1.8% of the subjects had concentrations ≥125 EU/mL, a figure similar to the 1.2% found in our study [29].

As already mentioned, serological assays measuring IgG antibodies against PT (which besides fimbriae is the only *B. pertussis*-specific antigen) are reliable and can be used in diagnostics and surveillance [21,23,30,31]. However, as antibodies to PT are also induced by vaccination [24], we analyzed the possible correlation of high PT antibody levels with antibody levels against the other four antigens. No statistical correlation was found between anti-PT antibodies and either of the four other antibodies, indicating that the high anti-PT levels in this ‘asymptomatic’ population were most likely the result of infection. 

In conclusion, the in-house pentaplex immunoassay is validated and is now available for performing a large serosurveillance/seroprevalence study on more than 3000 serum samples that have been collected in all age groups and in the three Belgian regions during 2013–2014.

## Figures and Tables

**Table 1:** Quality control of the pentaplex assay.

Analyte	LLOD (IU/mL)	Intra-Assay (CV%)	Inter-Assay (CV%)
Prn	0.2	2.15	2.38
FHA	0.032	2.47	2.60
PT	0.012	1.97	2.67
DT	0.00031	1.45	2.37
TT	0.00035	0.44	2.11

Lower limit of detection (LLOD); intra-assay and inter-assay coefficients of variation (CVs) of anti-diphtheria-tetanus-pertussis (DTP) IgG levels of the in-house reference serum.

**Table 2:** Geometric mean concentration (GMC) of anti-DT, anti-TT, anti-PT, anti-FHA and anti-Prn IgG antibodies according to age.

Antigen	age (year)	20	21	22	23	24	25	26	27	28	29	Adjusted
	*n*	29	50	54	63	70	77	64	92	110	61	
**DT**	GMC	**0.28**	**0.24**	**0.19**	**0.28**	**0.22**	**0.21**	**0.18**	**0.17**	**0.14**	**0.17**	**0.20**
	Lower 95% CI	0.16	0.16	0.12	0.19	0.15	0.15	0.12	0.12	0.10	0.11	0.13
	Upper 95% CI	0.49	0.37	0.31	0.41	0.32	0.30	0.27	0.24	0.18	0.28	0.29
**TT**	GMC	**0.91**	**0.50**	**0.51**	**0.61**	**0.64**	**0.69**	**0.75**	**0.56**	**0.64**	**0.49**	**0.63**
	Lower 95% CI	0.58	0.31	0.32	0.44	0.46	0.53	0.55	0.42	0.50	0.32	0.45
	Upper 95% CI	1.43	0.80	0.82	0.85	0.89	0.91	1.03	0.75	0.81	0.73	0.87
**PT**	GMC	**3.7**	**3.7**	**2.7**	**4.2**	**2.4**	**4.5**	**3.9**	**4.1**	**4.4**	**3.8**	**3.9**
	Lower 95% CI	1.9	2.4	1.6	2.9	1.6	3.1	2.6	3.1	3.1	2.4	2.6
	Upper 95% CI	7.5	5.9	4.5	6.0	3.8	6.7	5.9	5.6	6.3	6.2	5.8
**FHA**	GMC	**15.8**	**12.5**	**15.4**	**14.7**	**17.0**	**18.8**	**16.5**	**16.4**	**15.6**	**18.3**	**16.3**
	Lower 95% CI	8.1	8.5	10.1	10.8	12.2	14.2	12.0	12.2	12.3	12.8	11.7
	Upper 95% CI	30.8	18.3	23.4	20.0	23.8	24.9	22.6	22.1	19.8	26.2	22.7
**Prn**	GMC	**13.2**	**11.6**	**9.7**	**18.1**	**13.9**	**23.4**	**18.1**	**11.3**	**16.9**	**13.9**	**15.4**
	Lower 95% CI	6.3	7.3	5.9	11.3	9.5	15.8	12.1	7.8	12.0	8.4	10.1
	Upper 95% CI	27.8	18.5	15.8	29.1	20.4	34.8	27.2	16.5	23.8	23.0	23.6

GMC reported in IU/mL; CI: confidence interval; *n* = number of sera. The adjusted value was calculated, taking into account the number of samples in each age group.

**Table 3:** Number and percentage of subjects with serum concentrations below protection levels for diphtheria and tetanus and with serum levels indicative of pertussis infection during the last few years.

Antigen	IgG titer	E Flanders	W Flanders	Liège	Hainaut	Brussel	Bruxelles	Adjusted %
	*n*	120	127	122	61	123	116	
Diphtheria	<0.1 IU/mL	26 (22%)	23 (18%)	38 (31%)	18 (29%)	48 (39%)	60 (52%)	26.4
Tetanus	<0.1 IU/mL	10 (8.3%)	3 (2.4%)	12 (9.8%)	6 (9.8%)	14 (11.4%)	34 (29.3%	8.6
Pertussis (PT)	>50 IU/mL	8 (6.7%)	13 (10.2%)	4 (3.2%)	8 (13.1%)	6 (4.8%)	7 (6.0%)	8

**Table 4:** Geometric mean concentrations of anti-DT, anti-TT, anti-PT, anti-FHA and anti-Prn concentrations in subjects with anti-PT levels between 50 and 125 IU/mL (GMC A) and in subjects with anti-PT levels >125 IU/mL (GMC B).

GMC	DT	TT	PT	FHA	Prn
GMC A (*n* = 38)	0.34	1.06	74.9	64.4	66.4
Lower 95% CI	0.19	0.71	68.3	45.1	33.1
Upper 95% CI	0.59	1.59	82.2	91.9	133
GMC B (*n* = 8)	0.41	1.52	172	116	161
Lower 95% CI	0.17	0.74	135	53.4	38.4
Upper 95% CI	0.98	3.13	219	251	674

## References

[1] Theeten H., Hutse V., Hens N., Yavuz Y., Hoppenbrouwers K., Beutels P., Vranckx R., van Damme P. (2011). Are we hitting immunity targets? The 2006 age-specific seroprevalence of measles, mumps, rubella, diphteria and tetanus in Belgium. Epidemiol. Infect..

[2] Van der Wielen M., van Damme P., van Herck K., Schlegel-Haueter S., Siegrist C.A. (2003). Seroprevalence of Bordetella pertussis antibodies in Flanders (Belgium). Vaccine.

[3] Huygen K., Rodeghiero C., Govaerts D., Leroux-Roels I., Melin P., Reynders M., van Der Meeren S., van den Wijngaert S., Piérard D. (2014). Bordetella pertussis seroprevalence in Belgian adults 20–39 years old, anno 2012. Epidemiol. Infect..

[4] Prince H.E., Lape-Nixon M., Matud J. (2006). Evaluation of a tetraplex microsphere assay for Bordetella pertussis antibodies. Clin. Vaccine Immunol..

[5] Reder S., Riffelmann M., Becker C., Wirsing von Konig C.H. (2008). Measuring immunoglobulin g antibodies to tetanus toxin, diphtheria toxin, and pertussis toxin with single-antigen enzyme-linked immunosorbent assays and a bead-based multiplex assay. Clin. Vaccine Immunol..

[6] Van Gageldonk P.G., van Schaijk F.G., van der Klis F.R., Berbers G.A. (2008). Development and validation of a multiplex immunoassay for the simultaneous determination of serum antibodies to Bordetella pertussis, diphtheria and tetanus. J. Immunol. Methods.

[7] Van Gageldonk P.G., von Hunolstein H.C., van der Klis F.R., Berbers G.A. (2011). Improved specificity of a multiplex immunoassay for quantitation of anti-diphtheria toxin antibodies with the use of diphtheria toxoid. Clin. Vaccine Immunol..

[8] Stenger R.M., Smits M., Kuipers B., Kessen S.F., Boog C.J., van Els C.A. (2011). Fast, antigen-saving multiplex immunoassay to determine levels and avidity of mouse serum antibodies to pertussis, diphtheria, and tetanus antigens. Clin. Vaccine Immunol..

[9] Maertens K., Caboré R.N., Huygen K., Hens N., van Damme P., Leuridan E. (2016). Pertussis vaccination during pregnancy in Belgium: Results of a prospective controlled cohort study. Vaccine.

[10] Huygen K., Cabore R.N., Maertens K., Van D.P., Leuridan E. (2015). Humoral and cell mediated immune responses to a pertussis containing vaccine in pregnant and nonpregnant women. Vaccine.

[11] Luminex cookbook 2^nd^ Edition. http://info.luminexcorp.com/xmap-cookbook-2nd-edition-free-download.

[12] Waterboer T., Sehr P., Pawlita M. (2006). Suppression of non-specific binding in serological Luminex assays. J. Immunol. Methods.

[13] Bolstad N., Warren D.J., Nustad K. (2013). Heterophilic antibody interference in immunometric assays. Best. Pract. Res. Clin. Endocrinol. Metab.

[14] Amarasiri Fernando S., Wilson G.S. (1992). Studies of the 'hook' effect in the one-step sandwich immunoassay. J. Immunol. Methods.

[15] Wagner K.S., White J.M., Lucenko I., Mercer D., Crowcroft N.S., Neal S., Efstratiou A. (2012). Diphtheria in the postepidemic period, Europe, 2000–2009. Emerg. Infect. Dis..

[16] Detemmerman L., Rousseaux D., Efstratiou A., Schirvel C., Emmerechts K., Wybo I., Soetens O., Pierard D. (2013). Toxigenic *Corynebacterium ulcerans* in human and non-toxigenic *Corynebacterium diphtheriae* in cat. New Microbes. New Infect..

[17] European Centre for Disease Prevention and Control Rapid risk assessment: A fatal case of diphtheria in Belgium. http://ecdc.europa.eu/en/publications/_layouts/forms/Publication_DispForm.aspx?List=4f55ad51-4aed-4d32-b960-af70113dbb90&amp;ID=1458.

[18] WHO (2014). Immunization, Vaccines and Biologicals: Pertussis. http://www.who.int/immunization/monitoring_surveillance/burden/vpd/surveillance_type/passive/pertussis/en/.

[19] Crowcroft N.S., Pebody R.G. (2006). Recent developments in pertussis. Lancet.

[20] Crowcroft N.S., Stein C.A., Duclos P., Birmingham M. (2003). How best to estimate the global burden of pertussis?. Lancet Infect. Dis.

[21] Van der Zee A., Schellekens J.F., Mooi F.R. (2015). Laboratory Diagnosis of Pertussis. Clin. Microbiol. Rev..

[22] Baughman A.L., Bisgard K.M., Edwards K.M., Guris D., Decker M.D., Holland K., Meade B.D., Lynn F. (2004). Establishment of diagnostic cutoff points for levels of serum antibodies to pertussis toxin, filamentous hemagglutinin, and fimbriae in adolescents and adults in the United States. Clin. Diagn. Lab. Immunol..

[23] Guiso N., Berbers G., Fry N.K.H.Q., Riffelmann M., Wirsing von König C.H. (2011). EU Pertstrain group, What to do and what not to do in serological diagnosis of pertussis: Recommendations from EU reference laboratories. Eur J. Clin Microbiol. Infect. Dis..

[24] Wirsing von Konig C.H. (2014). Pertussis diagnostics: Overview and impact of immunization. Expert. Rev.Vaccines.

[25] De Greeff S.C., de Melker H.E., van Gageldonk P.G.M., Schellekens J.F.P., van der Klis F.R.M., Mollema L., Mooi F., Berbers G.A.M. (2010). Seroprevalence of Pertussis in the Netherlands: Evidence for Increased Circulation of *Bordetella pertussis*. PLoS ONE.

[26] Teepe J., Broekhuizen B., Ieven M., Loens K., Huygen K., Kretzschmar M., de Melker E., Butler C.L.P., Stuart B., Coenen S.G.V.T. (2015). Prevalence, diagnosis and disease course of pertussis in adults with acute cough in primary care. Br. J. General Pract..

[27] Braeye T., De Schrijver K., Piérard D., Huygen K. (2014). Kinkhoest. Infectieziekten bij kinderen die voorkomen kunnen worden door vaccinatie.Trends en Ontwikkelingen in België en de Gemeenschappen. 2012.

[28] Barkoff A.M., Grondahl-Yli-Hannuksela K., He Q. (2015). Seroprevalence studies of pertussis: what have we learned from different immunized populations. Pathog.Dis..

[29] Scott S., van der Sande M., Faye-Joof T., Mendy M., Sanneh B., Barry J.F., de Melker H., van der Klis F., van Gageldonk P., Mooi F. (2015). Seroprevalence of pertussis in the Gambia: evidence for continued circulation of bordetella pertussis despite high vaccination rates. Pediatr. Infect. Dis. J..

[30] Hallander H.O., Ljungman M., Storsaeter J., Gustafsson L. (2009). Kinetics and sensitivity of ELISA IgG pertussis antitoxin after infection and vaccination with *Bordetella pertussis* in young children. APMIS Authors J. Compil..

[31] Xing D., Markey K., Newland P., Rigsby P., Hockley J., He Q. (2011). EUVAC.NET collaborative study: evaluation and standardisation of serology for diagnosis of pertussis. J. Immunol. Methods.

